# Supplementary material for: Identification of photoperiod-induced specific miRNAs in the adrenal glands of Sunite sheep (Ovis aries)
Source: Front Vet Sci. 2022 Jul 22;9:888207. doi: 10.3389/fvets.2022.888207 (PMC9354845; doi:10.3389/fvets.2022.888207)
Supplement: Supplementary file 1 [file Data_Sheet_1.ZIP › Supplementary file/miRDeep2 file.zip!/miRDeep2 file/Novel_Structure/SPLP42A1/Novel_451.pdf]

Provisional ID	:	NC_019479.2_28214
Score total	:	1.8
Score for star read(s)	:	-1.3
Score for read counts	:	0
Score for mfe	:	1.5
Score for randfold	:	1.6
Score for cons. seed	:	
Total read count	:	25
Mature read count	:	25
Loop read count	:	0
Star read count	:	0

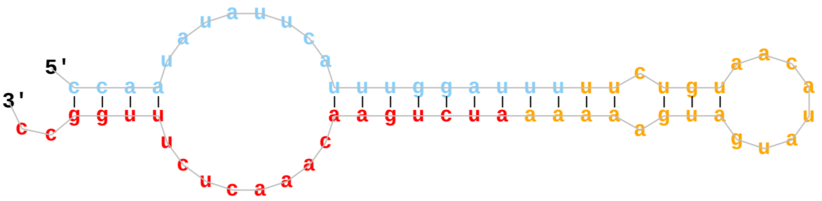

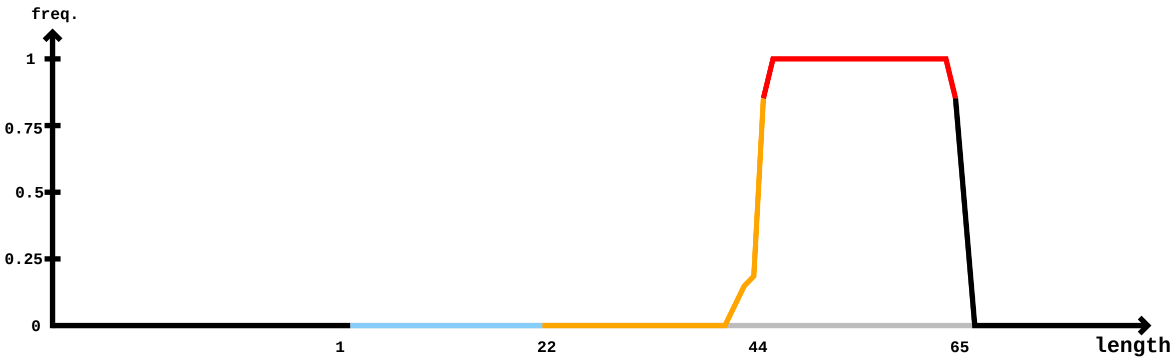

Star Mature

```
5'- gcaagaucuggaacaagguauuggguuggccaauauauucauuuggauuuuucuguaacauaugaugaaaaaaucugaacaaacucuuuggccaacccaauacuaugaggau   -3'   exp
    .............((.(((((((((((((((((........(((((((((((.(((........))).)))))))))))........))))))))))))))))).)).....    reads   mm   sample
    ......................................................................aaaaucugaacaaacuUuuuggc...................    2   1   seq
    .......................................................................aaaucugaacaaacucuuuggc...................    1   0   seq
    .......................................................................aaaucugaacaaacuUuuuggcc..................    1   1   seq
    ........................................................................aaucugaacaaacuUuuuggc...................    1   1   seq
    .........................................................................aucugaacaaacuUuuuggcc..................    18   1   seq
    ..........................................................................ucugaacaaacuUuuuggcca.................    4   1   seq
```
